# TWELVE YEARS OF CHANGES IN STATES’ ASSISTED LIVING REQUIREMENTS FOR DEMENTIA-SPECIFIC STAFF TRAINING

**DOI:** 10.1093/geroni/igz038.2006

**Published:** 2019-11-08

**Authors:** Paula Carder, Lindsey Smith, Seamus Taylor, Brian Kaskie, Kali S Thomas

**Affiliations:** 1 OHSU_PSU School of Public Health, Portland, Oregon, United States; 2 University of Iowa, Iowa City, Iowa, United States; 3 Brown University, Providence, Rhode Island, United States

## Abstract

We describe two categories of dementia-specific AL requirements: staff training and admission/discharge criteria. We reviewed current requirements for all states and the District of Columbia, and amendments made over 12 years. Current and historic regulations were collected and analyzed using policy surveillance and qualitative coding. Twenty-three states currently require dementia-specific training, and 22 require continuing education. Nearly all states (49) require administrators to complete dementia-specific training. Of these, 13 states specified 7 to 120 hours of dementia care training. Some states added pre-admission screening for cognitive impairment; a few require a dementia diagnosis for admission. We describe state variation longitudinally in direct care staff training requirements, including: number of training hours, training content, and use of examinations or other tests of knowledge, skills and abilities. In addition, we categorize changes in admission/discharge criteria over time, including the use of medical versus behavioral health symptoms.

